# Parasite OMICS, the grand challenges ahead

**DOI:** 10.3389/fpara.2022.995302

**Published:** 2022-08-05

**Authors:** Makedonka Mitreva

**Affiliations:** Department of Medicine, Washington University School of Medicine, St. Louis, MO, United States

**Keywords:** parasites, genomics, transcriptomics, proteomics, metabolomics, omics, applied genomics

## Background and introduction

Great progress has been made in parasitology as a result of the advancements made in both omics data generation and omics data interrogation approaches. The OMICS journey for many parasites started over 2 decades ago by generating and analyzing so called “expressed sequence tags” that profiled the most abundantly expressed transcripts in an organism at the time of sampling (Clifton and Mitreva, 2009). Improvements of these transcriptional profiles were technology-driven, starting with advancements in sample preparation and transitioning from traditional to more advanced sequencing platforms (from Sanger to Roche/454, and eventually the Illumina, PacBio and Oxford Nanopore platforms). With the new sequencing platforms, the amount of data that could be generated increases while the cost rapidly decreases, so the parasitology community continually adapts to the newest platforms and optimizes the methodology. As a result, some model species such as *Caenorhabditis elegans* have been resequenced repeatedly as each new platform became available, while new genomes for important parasites started being produced and improved. Parasites with smaller and simpler genomes were benefiting from these technologies earliest. The first eukaryotic genome to be published was the human malaria parasite *Plasmodium falciparum* in 2002, reporting a 23 Mb nuclear genome that encodes about 5,300 genes (Gardner et al., 2002). In 2005, the genomes of the three protozoan kinetoplastids were published: *Leishmania major* [33 Mb genome assembly containing 8,272 protein coding genes (Ivens et al., 2005)], *Trypanosoma cruzi* [67 Mb and ~12,000 genes (El-Sayed et al., 2005a)] and *T. brucei* [26 Mb and 9,068 genes (Berriman et al., 2005)], followed by a genome-wide comparison of these three members of the family *Trypanosomatidae* (El-Sayed et al., 2005b). By 2008, a 100 years after *Toxoplasma gondii* was initially described in Tunis by Nicolle and Manceaux (1908) (Kim and Weiss, 2008), a total of 12 *Toxoplasma* genomes were available including the *T. gondii* ME49 assembly (63 Mb and 8,032 genes). The first genome of a more complex multicellular eukaryotic parasite was that of the roundworm *Brugia malayi* [90 Mb and 11,500 genes (Ghedin et al., 2007)], published in 2007, 9 years later than the *C. elegans* genome (C. elegans Sequencing Consortium, 1998), and in 2009 the first flatworm *Schistosoma mansoni* [363 Mb, 11,809 genes (Berriman et al., 2009)] was published. In the next 7 years a total of 11 parasitic nematode genomes were published, when a “50 helminth genome initiative” was launched, resulting in the biggest data generation (45 new genomes) and comparative genome analysis published 5 years later (International Helminth Genomes Consortium, 2019). Genome size varied greatly from 42 to 700 Mb within nematodes, and from 104 to 1,259 Mb within platyhelminths, and this comparative study of the two major parasitic helminth phyla included 1.4 million genes and 81 helminth species.

As more genomes became available, it became clear that variations in parasite biology, physiology, mode of parasitism and tissue tropism were reflected in their genomes, as their varying size and complexity. The initially published genomes and transcriptomes revealed novel insights into the many parasites that occupy human, animal or plant hosts. However, there are many OMICS related challenges ahead of us that will require our immediate focus, including (but not limited to): (a) improving available genomes that are fragmented and/or inadequately annotated, and sequencing underrepresented species; (b) expanding on the types of OMICS data for many parasite species, and (c) applying the acquired OMICS-driven knowledge toward translational studies. These challenges are described below in more detail and are critically needed to better understand the complex biology of the parasites and advance their diagnosis, prevention, and control.

## Improving omics resources

Despite the progress in parasite genome production and improvement, the draft nature of many parasite genomes poses challenges for analysis and interpretation of the results, especially since post-genomic applications frequently require comparative genomics on a gene and single nucleotide level. Performing these analyses on draft genomes is inadequate due to gene fragmentations, gene model errors resulting from misassembly of allelic sequences as separate loci, the collapse of recently duplicated and diverged sequences into a single locus, and the large numbers of unordered contigs within scaffolds. The resulting incomplete or incorrect gene models for parasite species adversely affects many important analyses including (i) parasite drug target identification, since known or putative protein targets may not be properly annotated and therefore a drug's effect will not be predicted), (ii) homology modeling, for which accurate protein structure and active residue locations are critical for modeling drug interactions correctly, and (iii) the identification and classification of parasite-host interacting proteins, which are often difficult to annotate due to their diversification within and across species. In addition to the problems associated with gene model errors, highly fragmented genome assemblies negatively affect the analysis of selection signatures and identification of quantitative trait loci in experimental crosses and natural populations. Therefore, it is of pivotal importance that we continue to improve parasite assemblies and annotations using newly available technologies. Some examples of improvements include using HiFi PacBio long read sequencing, Oxford Nano Pore (ONT UL sequencing) and scaffolding with HiC linked reads, resulting in more contiguous assemblies. Direct reannotation of existing assemblies by single molecule PacBio mRNA sequencing can also be used to improve eukaryotic parasites genome annotation (Magrini et al., 2018) along with using multiple functional omics data and the newest available computational tools (Logan et al., 2020). While such improvements may not be as critically needed for some parasites that have smaller and/or simpler genomes, the genomic complexity of some species makes proper genome assembly and annotation very challenging; for example, the liver fluke *Fasciola* hepatica genome is 1.2 Gb, with 65% of the genome being repetitive. One of the big challenges ahead is that once the genome is published it is difficult to justify to funding agencies effort needed for improvement, so advocating for the importance of iterative improvements is essential. In addition to genome improvement, expanding the genomic resources for more parasitic species is equally important, as the genomes of some parasites of socio-economic, veterinary, or agricultural importance have yet to be sequenced.

## Expanding the omics repertoire

In addition to their genomes, advanced resources with the potential for major practical applications have been produced for many major parasites. Transcriptional profiles have been produced for whole parasites across their life cycles, in addition to parasite tissue-specific transcriptional profiles and transcriptional profiles of host responses to infection. In many cases, after these two data types (DNA and mRNA based) have become available, the sequencing data has been used to guide proteomics approaches including protein arrays and targeted and untargeted M/S proteomics to characterize peptides and catalog molecules involved in host-parasite interactions (such as excretory/secretory products of parasites) and to identify proteins bound by host antibodies following infection. Metabolomics approaches have also been used to understand gene pathway responses at a small molecule level, which can be used to validate the disruption of pathways thought to be targeted by drugs, or to identify changes in metabolism in the parasite under different conditions. These small biological molecules collectively known as the “metabolome” have a complex interplay in host-parasite interactions, and include molecules derived from/utilized by both the host and parasite, and is an emerging field of study (Whitman et al., 2021). mRNA expression profiling has recently started advancing from “bulk” RNAseq (whole-organism or whole-tissue) sequencing to single-cell RNA-seq (scRNAseq). While scRNAseq datasets are somewhat widely available for some model organisms, the field remains in its infancy for parasites. Similarly, host responses to infections at a single cell level have been documented for bacterial and viral infections, but for parasitology, the initial studies have focused on expression profiles of the parasites at a single cell level. For example, a recent paper used scRNAseq to identify tissue-specific cell types by profiling individual *T. brucei* trypanosomes from midgut, proventriculus, and salivary glands of infected tsetse flies (Howick et al., 2022). scRNAseq profiles have also been reported for infective and adult stage of *S. mansoni* (Diaz Soria et al., 2020; Wendt et al., 2020), two important stages of the parasite. Even though there is evidence for gene expression based on bulk or scRNAseq data, many of the genes are taxonomically restricted thus hypothetical. CRISPR-Cas9 genome editing approaches and RNAi screening, as reverse genetics methods that are particularly useful to functionally annotate taxonomically restricted parasite genes that do not have orthologs in other organisms. However, while these are robust and high-thruput in some parasites (e.g., kinetoplastids), they still pose a challenge for some parasites with more complex life cycle, and thus have been successfully performed for only a small number of genes in a few helminth species.

Parasites do not exist in isolation in the niche they occupy, so cross kingdom interactions involving the microbiome have also been studied over the last decade. Other OMICS data types have also started to emerge for the study of parasites, including spatial transcriptomics, epigenomics, glycomics, cytogenomics, immunomics to name a few.

## Applied omics—Translational aspects

As we have been entering into a post-genomic era, for some parasites, emerging OMICS technologies have been used to advance translational research and in practical applications. For example, traditional bulk genomic and transcriptomic sequencing and analyses have been biased toward the dominant genotype in samples, masking cell-to-cell variation and rare variants. For some species, this issue is being addressed with single-cell genome sequencing approaches, facilitating the quantification of genetic diversity and kinship in complex parasite populations and capturing *de novo* genetic variation (Dia and Cheeseman, 2021). Malaria transmission patterns are also being studied using whole genome sequencing of field isolates, and such approaches are starting to be applied to helminth infections, where efforts are underway to generate genetic tools to tackle post-treatment recrudescence of helminth infections, parasite emergence and spread, and drug resistance evolution. OMICS driven identification of targets essential for parasite survival has been proven to be correct in many cases, based on computational and experimental approaches [e.g., (Taylor et al., 2013; International Helminth Genomes Consortium, 2019; Tyagi et al., 2021; Ferreira et al., 2022)], and taking advantage of parasite specific molecular features to design drug-like compounds with higher potency compared to the host counterpart, and of taxonomically conserved targets to identify cross-clade efficacy against a broad spectrum of parasitic species (Tyagi et al., 2018). Such studies have closed the gap between genomics and actionable drug discovery, and progress toward lead identification and optimization for development of new antiparasitic drugs with broad spectrum activity and a novel mechanism of action. One of the challenges for global elimination of some parasitic infections such as onchocerciasis is the lack of highly sensitive, specific, and accurate diagnostic tools to detect adult female worms, and thus inform mass drug administration programs. Multi-omics profiling have provided datasets that could be mined for identification of candidates for improved diagnostics (Bennuru et al., 2016); indeed, some studies have mined the data for candidates for serodiagnosis (McNulty et al., 2015), and subsequent studies have fully characterized candidates for different infections (Curtis et al., 2021; Greene et al., 2022).

Different parasites in the ever evolving “OMICS ERA” are on different position of the OMICS spectrum, from resequencing the genomes of field isolates as an applied genomics approach for molecular surveillance, to the development of a systems biology approaches that can significantly contribute to more rational design for vaccines, therapeutics, and diagnostics.

## Author contributions

The author confirms being the sole contributor of this work and has approved it for publication.

## Funding

This study was supported by NIH Grants R01AI59450, R01AI144161, R01AI146353, and R01EY033195. The funding agencies had no role in the design, execution, or publication of this study.

## Conflict of interest

The author declares that the research was conducted in the absence of any commercial or financial relationships that could be construed as a potential conflict of interest.

## Publisher's note

All claims expressed in this article are solely those of the authors and do not necessarily represent those of their affiliated organizations, or those of the publisher, the editors and the reviewers. Any product that may be evaluated in this article, or claim that may be made by its manufacturer, is not guaranteed or endorsed by the publisher.
